# Novel biomarker genes which distinguish between smokers and chronic obstructive pulmonary disease patients with machine learning approach

**DOI:** 10.1186/s12890-020-1062-9

**Published:** 2020-02-03

**Authors:** Kazushi Matsumura, Shigeaki Ito

**Affiliations:** Scientific Product Assessment Center, R&D Group, Japan Tobacco Inc., 6-2 Umegaoka, Aoba-ku, Yokohama, Kanagawa 227-8512 Japan

**Keywords:** Gene expression, Cigarette smoke, Chronic obstructive pulmonary disease, Random forest, Classifier, Logistic regression, Computational scoring

## Abstract

**Background:**

Chronic obstructive pulmonary disease (COPD) is combination of progressive lung diseases. The diagnosis of COPD is generally based on the pulmonary function testing, however, difficulties underlie in prognosis of smokers or early stage of COPD patients due to the complexity and heterogeneity of the pathogenesis. Computational analyses of omics technologies are expected as one of the solutions to resolve such complexities.

**Methods:**

We obtained transcriptomic data by in vitro testing with exposures of human bronchial epithelial cells to the inducers for early events of COPD to identify the potential descriptive marker genes. With the identified genes, the machine learning technique was employed with the publicly available transcriptome data obtained from the lung specimens of COPD and non-COPD patients to develop the model that can reflect the risk continuum across smoking and COPD.

**Results:**

The expression levels of 15 genes were commonly altered among in vitro tissues exposed to known inducible factors for earlier events of COPD (exposure to cigarette smoke, DNA damage, oxidative stress, and inflammation), and 10 of these genes and their corresponding proteins have not previously reported as COPD biomarkers. Although these genes were able to predict each group with 65% accuracy, the accuracy with which they were able to discriminate COPD subjects from smokers was only 29%.

Furthermore, logistic regression enabled the conversion of gene expression levels to a numerical index, which we named the “potential risk factor (PRF)” index. The highest significant index value was recorded in COPD subjects (0.56 at the median), followed by smokers (0.30) and non-smokers (0.02). In vitro tissues exposed to cigarette smoke displayed dose-dependent increases of PRF, suggesting its utility for prospective risk estimation of tobacco products.

**Conclusions:**

Our experimental-based transcriptomic analysis identified novel genes associated with COPD, and the 15 genes could distinguish smokers and COPD subjects from non-smokers via machine-learning classification with remarkable accuracy. We also suggested a PRF index that can quantitatively reflect the risk continuum across smoking and COPD pathogenesis, and we believe it will provide an improved understanding of smoking effects and new insights into COPD.

## Background

Chronic obstructive pulmonary disease (COPD), a disorder characterized by reduced maximum expiratory flow and slow forced emptying of the lungs, is a common, costly, and preventable disease that has implications for global health [1]. Although cigarette smoke (CS) is a well-known risk factor for the development of COPD, smoking-related damage manifestations, such as airway wall thickening, loss of small airways functions, and emphysematous lung destruction, vary in individual smokers [2]. These heterogeneities of smoking-related manifestations lead to difficulty in investigating the risk continuum across smoking and COPD. Moreover, various next-generation products (NGPs), including e-cigarettes and heat-not-burn tobacco products, have been recently introduced in global markets [3, 4]. These NGPs can potentially reduce the harms associated with tobacco use because of their reduced yields of toxicants, which is attributable to the generation of aerosols without combusting tobacco leaves [5, 6], but the effects of long-term use of these NGPs on human health remain controversial [7, 8] despite previous non-clinical [9–12] and clinical studies [13–15]. Epidemiological analysis could be one of the solutions to estimate the realistic risk of the use of such products, but several years would be needed to reach a conclusion. Furthermore, epidemiological studies on a product-by-product basis would be difficult because new products are frequently introduced and customer choice would vary. Considering these issues together, rapid methodology for precisely predicting the potential risk of COPD is demanded to estimate the realistic impact of NGPs in comparison with combustible cigarettes.

Alternatives to animal testing have been introduced recently based on the principle of 3Rs: reduction, refinement, and replacement [16]. They have been also expected as rapid and precise risk assessment tools because of their high resemblance to in vivo situations [17]. In terms of investigating the effects of airborne materials such as CS, a three-dimensional (3D) cultured airway epithelial cell model that functionally differentiates through an air-liquid interface (ALI) culture is more representative, exhibiting a pseudostratified columnar epithelial structure with beating cilia as observed in the human airway [18, 19]. Our group also applied these in vitro alternative testing approaches to the investigation of biological responses to or prediction of the risks of acute or subchronic inhalation toxicity of CS [20–22]. In addition, the National Academy of Sciences [23] proposed a paradigm shift in toxicology from current animal-based testing toward the application of emerging technologies, including “-omics” technologies. This new paradigm would provide greater mechanistic insight into the mechanism by which many compounds affect human health [24]; therefore, omics technologies have also improved our understanding of the complex effects of CS [25–27]. Furthermore, these large-scale datasets may be well suited for computational methodology to develop risk prediction models [28]. However, the development of computational methodologies that can quantitatively assess human disease risk remains challenging issues.

The objective of the present study was to further understand of smoking effects and COPD pathogenesis. Among the existing omics technologies, we believe that the transcriptomic approach is one of the powerful tools because of the high quality of the data and availability of public available databases, such as ArrayExpress (https://www.ebi.ac.uk/arrayexpress/) and the GEO database (https://www.ncbi.nlm.nih.gov/geo/). Therefore, we first obtained the global transcriptomic profiles of CS exposure and COPD-related biological response inducers in ALI-cultured 3D human bronchial epithelial cells. However, the precise mechanism of action of CS exposure throughout the development of COPD has been unclear. CS-mediated oxidative stress is believed to be the uppermost biological event in respiratory tissues [29], and severe oxidative stress may lead to chronic inflammation and cellular DNA damage, as observed in the tissues of patients with COPD [30–32]. Thus, we exposed a commercially available 3D human airway epithelia reconstituted culture (MucilAir™) to the aqueous extract (AqE) of a reference cigarette and inducers of oxidative stress, cellular DNA damage, and inflammatory response. We hypothesized that the transcriptomes of tissues exposed to CS and those exposed to test substances possess valuable information related to COPD; therefore, we identified descriptive marker genes and their potential for reflecting the risk continuum across smoking and COPD pathogenesis. In this study, we developed an effective approach for new potential marker identification and estimation of disease risk using machine-learning techniques.

## Methods

### Test products

The 3R4F Kentucky reference cigarette (University of Kentucky, Lexington, KY, USA) was used as the representative conventional combustible cigarette and conditioned at 22 ± 1 °C and 60 ± 3% relative humidity for at least 48 h before use.

The oxidative stress inducers sodium hypochlorite (NaClO) and t-butylhydroquinone (tBHQ) were purchased from Wako Pure Chemical Industries, Ltd. (Osaka, Japan) [33, 34]. The DNA damage inducers cisplatin and bleomycin were purchased from Wako Pure Chemical Industries, Ltd. and Tokyo Chemical Industry Co., Ltd. (Tokyo, Japan) respectively [35, 36]. Human recombinant TNFα and IL-1β were purchased from Sigma-Aldrich (St Louis, MO, USA) and used as inflammatory response mediators.

### Cell culture

MucilAir human bronchial epithelial cultures were purchased from Epithelix Sàrl (Geneva, Switzerland). The cultures were composed of epithelial cells from 61-year-old Caucasian male non-smoker in MucilAir™ (batch number: MD053701). The MucilAir™ tissues, cultured in 24-well-sized Transwell inserts (Corning, Corning, NY, USA), were placed into 24-well plates (Corning) with 700 μL of MucilAir™ culture medium (Epithelix Sàrl) upon arrival. The tissues were incubated at 37 °C in a 5% CO_2_ atmosphere for more than 10 days for acclimation before starting exposure [37]. The medium was changed every 2–3 days.

### Preparation of the AqE of cigarette smoke

The ISO Intense smoking regimen (ISO 20778: a 55-mL bell-shaped puff taken over 2 s, repeated every 30 s with blocking of filter ventilation [38]) was used for smoking 3R4F cigarettes. The AqE was prepared by bubbling the mainstream aerosol generated from 3R4F cigarettes through PneumaCult-ALI without the supplements (StemCell Technologies, Vancouver, BC, USA). Two 3R4F cigarettes were smoked in one smoking cycle to a butt length of 35 mm with RM20H (Borgwaldt, Hamburg, Germany), and approximately 2.0 cigarettes of 3R4F smoke were bubbled into 15 mL of ice-cold PneumaCult-ALI without the supplements. The supplement of PneumaCult-ALI medium was added immediately before mixing with MucilAir™ culture medium in accordance with the manufacturer’s instructions. The AqE was diluted with MucilAir™ culture medium to concentrations of 0.5, 1.0, and 2.0 cigarettes/L.

### Experimental design of the exposure studies

The exposure studies were performed using each test substance and the AqE of 3R4F smoke. MucilAir™ tissue was exposed to each test substance for 4 or 24 h at the following concentrations: 8 and 16 μM (cisplatin), 50 and 100 μg/mL (bleomycin), 0.7 and 1.4 mM (NaClO), 20 and 40 μM (tBHQ), 20 and 40 ng/mL (TNFα), and 20 and 40 ng/mL (IL-1β). MucilAir™ tissue was exposed to the AqE of 3R4F smoke for 4 and 24 h at 0.5, 1.0 and 2.0 cigarettes/L. Non-treated MucilAir™ tissue was used as a control.

### Transcriptomic analysis using mRNA extracted from MucilAir tissues

Total RNA was isolated from three tissue cultures at each time point using RNeasy (Qiagen, Hilden, Germany). The RNA quality of the samples was rated according to the RNA integrity number using an Agilent 2100 Bioanalyzer (Agilent Technologies, Santa Clara, CA, USA). Microarray analysis was conducted by Takara Bio, Inc. (Shiga, Japan) using Human Genome U133 Plus 2.0 arrays (Affymetrix, Santa Clara, CA, USA). Raw data were summarized using the GC-Robust Multiarray Average in GeneSpring Version 14.9.1 (Agilent Technologies). Data with a normalized intensity value below the 20th percentile and coefficient of variation ≥50% were filtered out. The filtered list was analyzed using a moderated *t*-test, and multiple testing correction of the *t*-test *p*-values was performed using the Benjamini-Hochberg FDR [39] to detect significant differences at an FDR-corrected *p* < 0.05 between the exposure groups and the controls. These normalization processes were separately performed for both the AqE- and test substance-exposed groups to remove genes that exhibited high coefficients of variation. Genes exhibiting significant changes (FDR-corrected *p* < 0.05 and |fold change| > 1.5 in the AqE-exposed group and FDR-corrected *p* < 0.05 in the test substance-exposed groups) were defined as DEGs. Hierarchical clusters and Spearman’s rank correlation heatmap were generated using GeneSpring Version 14.9.1. Transcriptomic data are available in ArrayExpress at accession number E-MTAB-7992.

### Identification of descriptive genes from in vitro exposure study

To identify the descriptive genes related to COPD, we analyzed the transcriptomic data from the in vitro exposure studies as follows: (1) To identify the CS-inducible genes, we analyzed the gene expression profiles of MucilAir™ exposed to the AqE from 3R4F smoke for 4 h at three different concentrations (0.5, 1.0, and 2.0 cigarettes/L). We compared upregulated and downregulated genes separately, and identified 25 commonly upregulated DEGs and 25 commonly downregulated DEGs (|fold change| > 1.5, false discovery rate [FDR]-corrected *p* < 0.05) from the Venn diagrams. (2) To identify the genes associated with COPD-related biological processes, we compared the gene expression profiles of each inducer. We extracted DEGs in each test substance (cisplatin, bleomycin, NaClO, tBHQ, TNFα, and IL-1β)) at each dose (low and high) and at each time-point (4 or 24 h), and identified common DEGs between 4 and 24 h exposure at the same dose for each test substance (time-independently perturbed DEGs). Subsequently, we integrated all the time-independently perturbed DEGs in each test substance, and investigated the gene expression profile using hierarchical clustering analysis. Finally, we extracted the dose-independently perturbed DEGs. (3) We compared the up- and downregulated DEGs identified in steps (1) and (2) in a Venn diagram, and ultimately identified 15 descriptive genes. Figure 2 is a graphical summary of the results.

### Data processing and classification analysis of public microarray datasets

Three previously published datasets for bronchia (E-MTAB-1690) and small airways (E-GEOD-20257 and E-GEOD-8545) were obtained from EMBL ArrayExpress. Raw data were summarized using GC-Robust Multiarray Average in GeneSpring Version 14.9.1. The summary of sample information is shown in Table 1. The platform for all microarray data was Human Genome U133 Plus 2.0 Array, which we also used. These datasets included 68 non-smokers, 88 smokers, and 48 COPD subjects. These subjects were used to calculate the multi-classification accuracy using the identified genes with the RF algorithm and develop the computable model using logistic regression analysis. Multi-classification analysis using the RF algorithm were performed in the R 3.5.2 statistic environment with “caret” packages [40]. The accuracy was calculated using RF with 5-fold cross-validation, repeated on 100 times independently. One-way ANOVA followed by Tukey’s honest significant difference post-hoc test was performed to compare significant differences (*p* < 0.05) between groups using R software with “multcomp” packages [41].

**Table 1:** Overall summary of the publicly available datasets

Study name	Study samples	Age	Sex	Sample type
E-MTAB-1690	14 NS, 27 SMK, and 21 COPD	51.9 ± 8.69	53 male, 9 female	Respiratory tract
E-GEOD-20257	36 NS, 43 SMK, and 9 COPD	42.8 ± 10.9	61 male, 27 female	Small airway
E-GEOD-8545	18 NS, 18 SMK, and 18 COPD	45.7 ± 7.18	41 male, 13 female	Small airway

*NS* non-smokers, *SMK* smokers, *COPD* COPD subjects

### Individual COPD risk score prediction modeling

Normalized expression values of samples were used to calculate the individual COPD potential risk score, named the PRF index, as follows:

Stepwise logistic regression was performed in the R statistical environment to extract the characteristic genes of smokers and COPD subjects. We then developed a prediction model with these genes using logistic regression. The equations for estimating probabilities of smoker (*P*_*SMK*_) and COPD (*P*_*COPD*_) were as follows:

$$ \mathrm{logit}\left({p}_{SMK}\right)=\ln \left(\frac{p_{SMK}}{1-{p}_{SMK}}\right)={C}_{NS\mid SMK}+{\beta}_1{m}_1+{\beta}_2{m}_2+\cdots +{\beta}_i{m}_i $$

$$ \mathrm{logit}\left({p}_{COPD}\right)=\ln \left(\frac{p_{COPD}}{1-{p}_{COPD}}\right)={C}_{SMK\mid COPD}+{\gamma}_1{n}_1+{\gamma}_2{n}_2+\cdots +{\gamma}_j{n}_j $$

where *C*_*NS|SMK*_ and *C*_*SMK|COPD*_ denote the intercepts of each prediction model with the genes selected by comparing non-smokers to smokers and smokers to COPD subjects, respectively, *m*_*i*_ or *n*_*j*_ is the normalized expression value of the *i*^*th*^ or *j*^*th*^ gene, respectively, and *β*_*i*_ or *γ*_*i*_ denote the regression coefficient of the *i*^*th*^ or *j*^*th*^ gene, respectively. The probabilities *P*_*smk*_ and *P*_*COPD*_ were then used to compute the PRF index as follows:

$$ \mathrm{PRF}=\frac{p_{SMK}\times {p}_{COPD}}{1-\left({p}_{SMK}\times {p}_{COPD}\right)} $$

### Statistical analysis

Tukey–Kramer multiple comparison analysis was conducted using JMP ver. 14.2.0 (SAS Institute, Cary, NC, USA) to compare significant differences (*p* < 0.05) between groups.

## Results

### Differential analysis of the gene expression profiles related to the AqE of 3R4F smoke and stress inducers

**Fig. 1:**
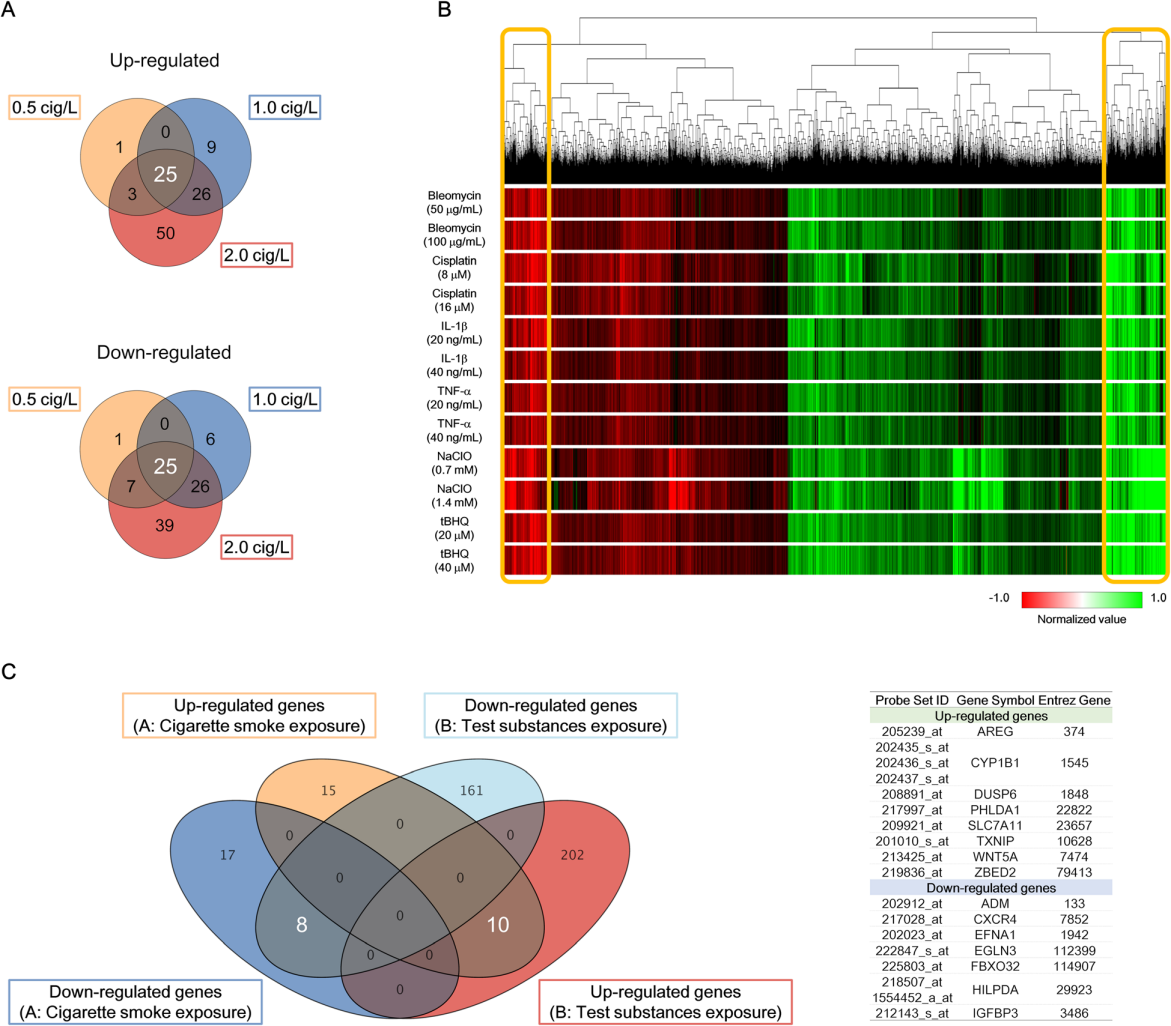
Identifying descriptive marker genes from in vitro exposure studies. **a** Venn diagram of up- and downregulated genes (|fold change| > 1.5, false discovery rate-corrected *p* < 0.05) following exposure to the aqueous extract of 3R4F smoke at a concentration of 0.5, 1.0, or 2.0 cigarettes/L. **b** Hierarchical clustering analysis with time-independently differentially expressed genes (DEGs) (false discovery rate-corrected *p* < 0.05) following exposure to each test substance. The orange box denotes up- or downregulated gene clusters. **c** Venn diagram of up- and down-regulated DEGs derived from (**a**) cigarette smoke exposure studies and (**b**) test substances exposure studies. Identified 15 descriptive genes is summarized in the right table of the Venn diagram. Cig, cigarettes

**Fig. 2:**
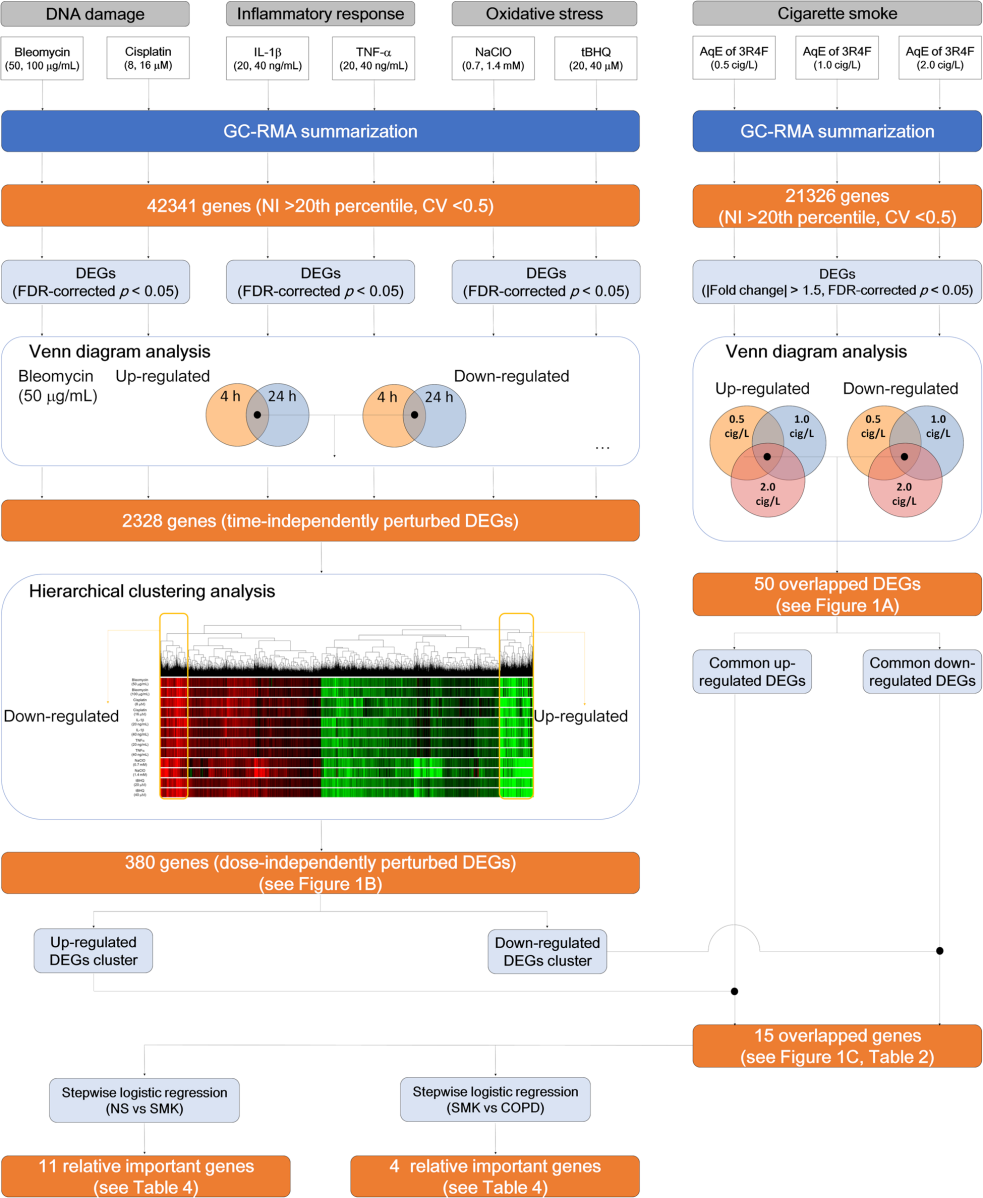
Schematic diagram for identifying descriptive marker genes. The process for identifying descriptive marker genes for multi-classification and stepwise logistic regression analysis. Cig, cigarettes; CV, coefficient of variation; NI, normalized intensity value; NS, non-smokers; SMK, smokers; COPD, chronic obstructive pulmonary disease subjects

We analyzed the gene expression profiles of MucilAir™ exposed to the AqE of 3R4F smoke for 4 h at three different concentrations (0.5, 1.0, and 2.0 cigarettes/L) and identified 50 dose-independently up- and downregulated differentially expressed genes (DEGs) (|fold change| > 1.5, false discover rate [FDR]-corrected *p* < 0.05) (Fig. 1a). We also obtained the gene expression profile for each inducer. We extracted time-independently perturbed DEGs, and these DEGs were subjected to hierarchical clustering analysis to identify dose-independently perturbed DEGs (Fig. 1b). The analysis revealed concentrated gene clusters containing highly up- and downregulated genes. Consequently, we merged DEGs in the AqE of 3R4F smoke and from the stress inducers, and identified 15 genes commonly up- or downregulated by the AqE of 3R4F smoke and inducers (Fig. 1c). The process of gene identification is summarized in Fig. 2, and these genes and their known functions and confirmation of their association with COPD or lung function via literature reviews using PubMed are summarized in Table 2.

**Table 2:** Known function and association with chronic obstructive pulmonary disease (COPD) for identified genes

Gene	Known function of the gene product	References (PMIDs)
Upregulated genes		
AREG	Member of the EGF family, which interacts with the EGF/TGF-alpha receptor to promote the growth of normal epithelial cells.	Stolarczyk M, et al. (27561911), Wang J, et al. (30291869)
CYP1B1	Member of the cytochrome P450 superfamily of enzymes. High expression is induced by cigarette smoke exposure.	Liu C, et al. (29110844), Slowikowski BK, et al. (28858732)
DUSP6	Dual-specificity protein phosphatase subfamily. It negatively regulates MAPK superfamily proteins, which are associated with cellular proliferation and differentiation.	–
PHLDA1	Proline–histidine-rich nuclear protein that might play an important role in the anti-apoptotic effects of insulin-like growth factor-1.	–
SLC7A11	Sodium-independent, high-affinity exchange of anionic amino acids with high specificity for the anionic forms of cystine and glutamate.	–
TXNIP	Thioredoxin-binding protein that inhibits the antioxidative function of thioredoxin, resulting in the accumulation of ROS and cellular stress.	–
WNT5A	Wnt family member 5A, ligand for members of the frizzled family of seven-transmembrane receptors.	Koopmans T, et al. (27468699), Baarsma HA, et al. (27979969)
ZBED2	Zinc finger BED-type containing 2.	–
Downregulated genes		
ADM	Preprohormone with several functions, including vasodilation, regulation of hormone secretion, promotion of angiogenesis, and antimicrobial activity.	Xu P, et al. (14720432), Meng DQ, et al. (24962223)
CXCR4	CXC chemokine receptor specific for stromal cell-derived factor-1.	Weigold F, et al. (29566745), Karagiannis K, et al. (28804668)
EFNA1	Member of the ephrin family. Its target receptors comprise the protein-tyrosine kinases, and it has been implicated in mediating developmental events.	–
EGLN3	Hypoxia-inducible factor. Essential for the hypoxic regulation of neutrophilic inflammation and it has crucial role in DNA damage response.	–
FBXO32	Fbox protein that functions in phosphorylation-dependent ubiquitination and subsequent proteasomal degradation.	–
HILPDA	Hypoxia-inducible lipid droplet-associated protein. Stimulates cytokine expression and enhances cell growth and proliferation.	–
IGFBP3	Insulin-like growth factor binding protein family. It prolongs the half-life of IGFs and alters their interaction with cell surface receptors.	–

The cited references describe the confirmation of the association of the selected genes with COPD or lung function, which were obtained by reviewing the literature using PubMed ((“COPD” OR “Lung Function”) AND “name of each selected gene”)

### Predictive performance of smoking and COPD status with identified genes

**Fig. 3:**
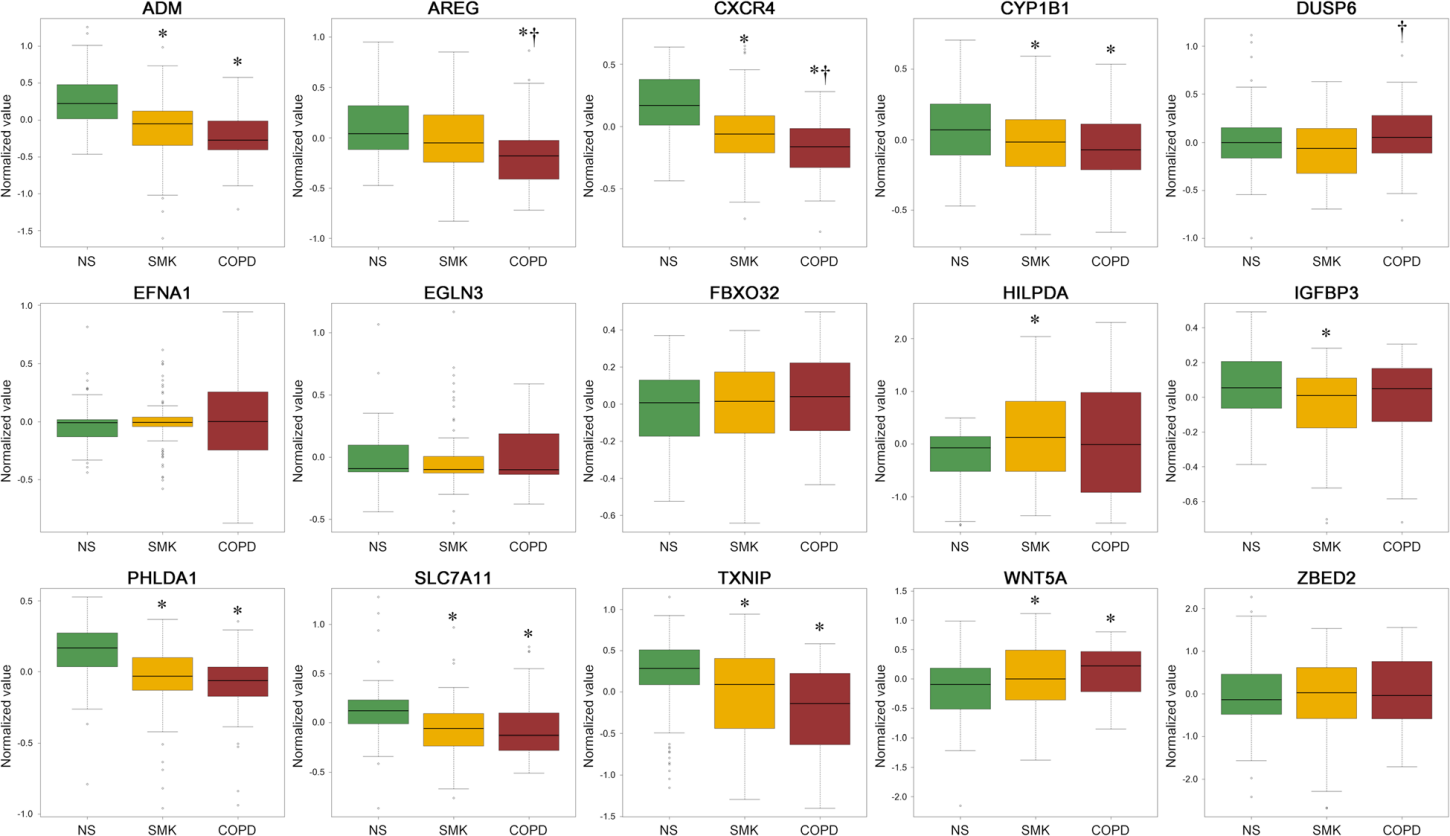
Expression value of identified genes in publicly samples. The box plot presents the normalized expression values of the 15 identified genes in publicly available samples for non-smokers (green), smokers (yellow), and chronic obstructive pulmonary disease (COPD) subjects (red). The box plot presents the median (line) and 25th and 75th percentiles (box); the whiskers are the 5th and 95th percentiles; and the outliers are denoted by open circles. One-way ANOVA with subsequent Tukey’s honest significant difference post-hoc analysis revealed differences between NS and SMK or COPD (*p < 0.05) and between SMK and COPD (†p < 0.05). NS, non-smokers; SMK, current smokers; COPD, COPD subjects

The expression levels of the 15 identified genes were compared using publicly available microarray datasets of non-smokers, smokers, and COPD subjects (Fig. 3). Compared with the findings in non-smokers, the expression levels of ADM, AREG, CXCR4, CYP1B1, PHLDA1, SLC7A11, TXNIP, and WNT5A were significantly different in COPD subjects. In addition, compared with the findings in smokers, the expression levels of AREG, CXCR4, and DUSP6 were different in COPD subjects. We then predicted the accuracy of classification of smokers and COPD subjects using these 15 genes with the random forest (RF) classification algorithm. The 5-fold, 100 times repeated cross-validation accuracy with the identified genes outperformed that for genes described by Bosse [42] based on sensitivity and specificity values (Table 3).

**Table 3:** Multi-classification analysis with random forest (5-fold cross-validation repeated 100 times independently)

Gene set	Original				Published				Extended			
Pred./Truth	NS	SMK	COPD	True rate	NS	SMK	COPD	True rate	NS	SMK	COPD	True rate
NS	25.5	5.8	1.0	0.77	16.1	10.2	4.2	0.48	19.7	8.8	2.4	0.59
SMK	7.2	32.6	15.8	0.76	15.0	25.9	15.0	0.60	13.0	30.4	15.0	0.71
COPD	0.6	4.8	6.7	0.29	2.2	7.0	4.3	0.18	0.6	4.0	6.2	0.26

Classification analysis with random forest was performed using the identified 15 genes (Original) and previously published genes, including genes cited in > 10 (Published) or > 6 publications (Extended)

*NS* non-smokers, *SMK* smokers, *COPD* COPD subjects

### Computable scoring method of potential COPD risk

**Fig. 4:**
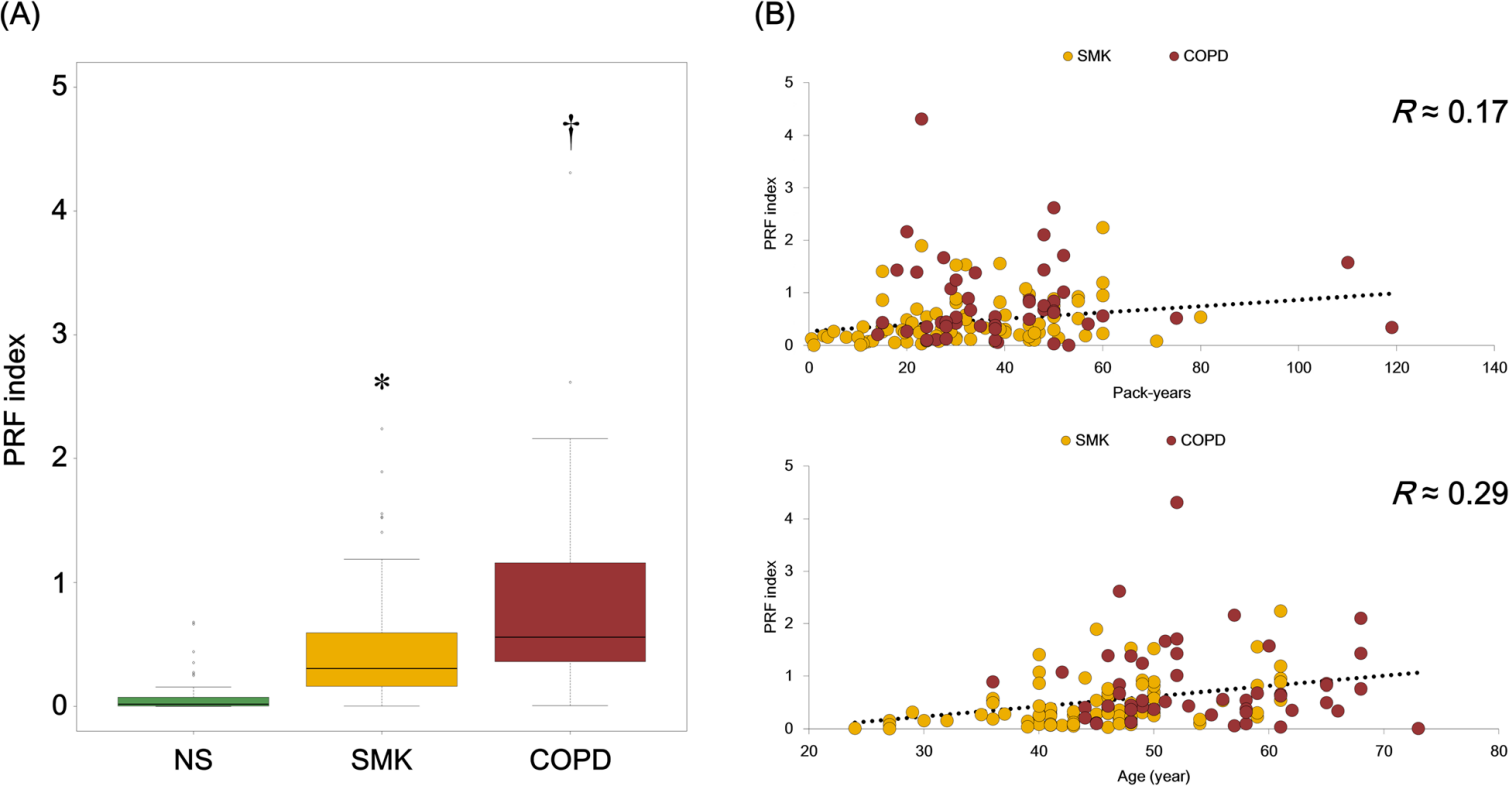
Potential risk factor calculation with publicly samples. **a** The box plot showing the potential risk factor (PRF) indices of non-smokers (green), smokers (yellow), and chronic obstructive pulmonary disease (COPD) subjects (red) in publicly available samples. The box plot presents the median (line) and 25th and 75th percentiles (box); the whiskers are the 5th and 95th percentiles; and the outliers are denoted by open circles. One-way ANOVA with subsequent Tukey’s honest significant difference post-hoc analysis revealed differences between NS and SMK (*p < 0.05) and between SMK and COPD (†p < 0.05). **b** The correlations of the PRF indices with pack-years and age in smokers and COPD subjects. The Pearson correlation coefficient (*R*) is shown in upper right of each image. NS, non-smokers; SMK, smokers; COPD, COPD subjects

To investigate the utility of the identified genes for assessing the potential risk of COPD, we compared the potential risk factor (PRF) indices of non-smokers, smokers, and COPD subjects. Because of the similar gene expression pattern between smokers and COPD subjects, a stepwise logistic regression model was applied to extract the characteristic genes of smokers and COPD subjects. We finally identified 11 genes (ADM, AREG, CXCR4, EFNA1, EGLN3, FBXO32, HILPDA, IGFBP3, SLC7A11, TXNIP, and WNT5A) as potential descriptive marker genes for smokers and 4 genes (AREG, DUSP6, EFNA1, and TXNIP) as potential descriptive marker genes for COPD subjects. We then calculated the logistic regression equation using the 11 and 4 genes, and the calculated parameters are summarized in Table 4. We then calculated the respective PRF indices of non-smokers, smokers, and COPD subjects to verify the validity. As expected, the highest PRF index was recorded in COPD subjects (0.56 at the median), followed by smokers (0.30) and non-smokers (0.02) (Fig. 4a). We also analyzed the correlations between the PRF index and both pack-years and age (Fig. 4b), and found that there was little correlation between the PRF index and pack-years (*R* ≈ 0.17), and between the PRF index and age (*R* ≈ 0.29).

**Table 4:** The parameters calculated via stepwise logistic regression analysis

	Estimate	Std. Error	Z value	Pr(>|Z|)
(Intercept) NS|SMK	1.6715	0.4229	3.953	7.73E−05
ADM	−2.2568	1.0247	−2.202	0.027641
AREG	2.0152	1.0407	1.936	0.052820
CXCR4	−3.1177	1.4308	− 2.179	0.029336
EFNA1	3.7889	1.9572	1.936	0.052882
EGLN3	−4.0571	1.7627	−2.302	0.021357
FBXO32	−3.8824	2.4113	−1.610	0.107376
HILPDA	3.2193	0.8100	3.974	7.06E−05
IGFBP3	−8.2992	2.3747	−3.495	0.000474
SLC7A11	−3.5355	1.2516	−2.825	0.004730
TXNIP	−5.6745	1.4851	−3.281	1.33E−04
WNT5A	2.7391	0.8231	3.328	8.75E−04
(Intercept) SMK|COPD	−0.8555	0.2144	−3.990	6.61E−05
AREG	−1.3039	0.7058	−1.847	0.06469
DUSP6	1.4688	0.6254	2.349	0.01885
EFNA1	2.3861	0.9058	2.634	0.00843
TXNIP	−0.8847	0.5167	−1.712	8.69E−02

## Discussion

In this study, we utilized a 3D cultured bronchial epithelial tissue model, which is expected to be one of the alternative models to animal testing. We conducted exposure studies using the AqE of 3R4F smoke and inducers of oxidative stress, DNA damage, and inflammatory responses because these are considered the earliest key events for chronic inflammatory lung diseases [29–32]. To identify potential descriptive marker genes, we extracted commonly up- and down-regulated genes from the transcriptomes of tissues exposed to those test substances (Fig. 2). ADM, AREG, CXCR4, CYP1B1, DUSP6, EFNA1, EGLN3, FBXO32, HILPDA, IGFBP3, PHLDA1, SLC7A11, TXNIP, WNT5A, and ZBED2 were identified as commonly perturbed genes, and 10 of these genes, as well as their coding proteins, had not previously been identified as biomarkers for chronic inflammatory lung disease or associated with lung function (Table 2). In addition, these 15 genes were highly correlated with each other (Additional file 1: Figure S1), suggesting that they are perturbed by the same or similar mechanisms. To verify the association of these 15 genes with COPD pathology, we performed RF-based multi-classification to discriminate COPD subjects, smokers, and non-smokers using publicly available transcriptomic data (Table 1). This model with the 15 genes clarified patient status with marginally higher accuracy than known COPD-associated genes [42], suggesting that the 15 genes, including newly identified potential marker genes, are closely associated with COPD status. These newly identified biomarkers are related to proliferation (DUSP6 [43], EFNA1 [44], IGFBP3 [45], and PHLDA1 [46]), hypoxia (EGLN3 [47] and HILPDA [48]), redox homeostasis (SLC7A11 [49] and TXNIP [50]), and epithelial-mesenchymal transition (FBXO32 [51]) (Table 2). Among them, the expression levels of AREG, CXCR4 and DUSP6 were significantly different between non-smokers and COPD subjects, and these genes are known to be associated with EGFR signaling, which plays a key role in the pathogenesis of COPD [52]. AREG, an EGFR ligand generated by the ADAM17-mediated shedding of pro-AREG proteins, stimulates the transcription of inflammatory mediators in bronchial epithelial cells [53]. Moreover, recent research illustrated that AREG-mediated IL-6 secretion is enhanced in differentiated bronchial cells from patients with COPD compared with the findings in cells from subjects without COPD [54, 55]. CXCR4 is associated with the recruitment of lymphocytes to disease lesions [56]. The mRNA levels of the CXCR4 ligand SDF-1 are reduced in mesenchymal stem cells (MSCs) derived from bone marrow, suggesting an impairment of the migratory capacity of MSCs. MSC migration to disease lesions plays crucial roles in anti-inflammatory effects and tissue repair [57, 58]. The publicly available transcriptomic data used in this study were obtained from lung biopsies; however, downregulation of CXCR4 in COPD subjects implies attenuation of MSC recruitment, thereby eventually accelerating inflammation and tissue destruction. Although the direct relationship between DUSP6 and COPD has not yet been reported, several advanced studies demonstrated that activation of EGFR induces DUSP6, which regulates EGFR signaling via specific ERK1/2 inhibition [59]. Therefore, the observation of DUSP6 upregulation in COPD subjects in this study implies constitutive activation of the EGFR signaling pathway. Taken together, these three genes extracted from the transcriptome of in vitro tissues may be associated with COPD pathogenesis via the EGFR signaling pathway, and they are expected as novel markers of COPD.

Although the 15 genes were able to predict non-smokers, smokers, and COPD subjects with high accuracy, the result clearly revealed that it is difficult to discriminate COPD subjects from smokers (Table 3). Therefore, we provide the PRF index model based on a logistic regression method to distinguish COPD subjects from smokers. This approach enabled the conversion of gene expression levels to a numeral index named the PRF index (see the formula in the Materials and Methods section). Logistic regression is used frequently in clinical trials to calculate the odds ratio when the risk ratio cannot be obtained directly [60]. The PRF index is also based on the concept of odds ratios, which indirectly estimate the risk ratio of CS exposure. Because the gene expression profiles of smokes and COPD subjects were similar, we first performed stepwise elimination of the 15 extracted genes to identify important variables. We selected 11 genes as important for distinguishing non-smokers from smokers, and 4 genes for distinguishing smokers from COPD subjects. Interestingly, 3 out of 4 genes for distinguishing smokers from COPD subjects (AREG, EFNA1, and TXNIP) were also marker genes for distinguishing non-smokers from smokers (Table 4). AREG was considered to be associated with EGFR signaling pathway activation as described. EFNA1 encodes a member of the ephrin family, ephrin A1. Advanced studies suggest that these proteins play an important role in inflammation through NF-κB signal activation [61]. Thioredoxin-interacting protein (TXNIP) reduces the anti-oxidative function of thioredoxin by binding to its redox-active cysteine residues [62, 63]. The expression level of EFNA1 increased in smokers compared with non-smokers, and was higher in COPD subjects than in smokers (Fig. 3). On the other hand, the expression level of TXNIP decreased in smokers compared with non-smokers, and was lower in COPD subjects than in smokers. These data suggest that those gene expression levels could provide an important means of distinguishing between smokers and COPD subjects. The PRF index was then calculated using the normalized expression values of the selected genes, the estimated intercept, and the regression coefficient of each gene. The PRF indices of smokers and COPD subjects were significantly different from that of non-smokers (Fig. 4a). Because the ages and pack-years differed significantly between the smokers and COPD subjects (Additional file 2: Figure S2A), and were moderately correlated (Additional file 2: Figure S2B), we analyzed the correlations of the PRF indices and the expression values of the 15 identified genes with age and pack-years. AREG and TXNIP exhibited weak correlations with both pack-years (Additional file 3: Figure S3) and age (Additional file 4: Figure S4). However, the other genes exhibited little correlation, and notably, there were very weak correlations between the PRF indices and those factors. This suggests that a combination of several genes could appropriately reflect the risk continuum across smoking and COPD pathogenesis, and also, each individual genes used in the PRF index model may provide further understanding of smoking effects and new insights into COPD.

Although the PRF index does not reflect future COPD risk, and is incapable of diagnosing COPD severity in individuals, the model may have a potential to compare the toxicity of various tobacco products in in vitro study based on the COPD-related biological responses. We also calculated the PRF index using MucilAir™ samples exposed to the AqE of 3R4F smoke for 4 and 24 h (Additional file 5: Figure S5). Although dose-dependent increases of the PRF index were observed, the PRF index for the lowest concentration of the AqE of 3R4F smoke was less than 1.0, indicating a lower risk than observed for the air-exposed control group. Because the pathological or morphological changes in smokers or patients with COPD could be caused by habitual cigarette smoking, we must examine the variability of the PRF index in a repeated long-term CS exposure study in a future analysis to validate the PRF index using in vitro experimental datasets for prospective risk estimations. In addition, it is also a reasonable next step to calculate the PRF index in a study comparing exposure to NGP vapor and conventional combustible cigarette smoke to demonstrate the usefulness of the index for the potential assessment of the relative toxicity based on the COPD-related biological responses.

We believed our model and PRF index are useful for the discrimination of non-smokers, smokers, and COPD subjects, but there are some limitations, which must be considered further. (i) Because cigarette smoking can have acute and eventually chronic effects, the smoking status of the subjects is an important consideration with regard to the gene signature (e.g., the gene expression profiles would be different between smokers with COPD and former smokers with COPD). However, we only found a clear description of the smoking status of the subjects in the E-MTAB-1690 study [64–66]. Therefore, it is possible that our model ignored the factors related to acute phase effects in the COPD subjects. (ii) Eight substances, focusing on three biological events, were used to identify COPD-associated biomarker genes. Because COPD is a complex disease, other important biological perturbations such as apoptosis and autophagy are involved. Gene expression profiles obtained in additional exposure studies using the inducers of such biological events would increase the plausibility of potential biomarker genes. (iii) We utilized microarrays to analyze gene expression profiles in this study; however, next-generation sequencing could potentially permit a more comprehensive analysis of RNA expression profiles including non-coding RNAs. As such, room for improvement of our methodology remains, but our present approach suggests that mechanism-based large-scale dataset generation combined with computational analyses is useful for biomarker identification and risk estimation using the identified biomarker genes.

## Conclusion

Our results strongly suggest that the combination of large-scale datasets and computational modeling represents a powerful approach for identifying novel biomarkers to further understand the smoking effects and providing new insights into COPD. Considering that the selected genes were originally identified in an in vitro exposure study, the application of PRF scoring for prospective toxicity of combustible CS and comparisons with NGPs in a repeated long-term exposure study are conceivable next steps.

## Supplementary information

**Additional file 1: Figure S1.** Spearman’s correlation coefficients analysis. Hierarchical clustering of the Spearman’s correlation coefficients of the 15 identified genes. Normalized intensity values following exposure to the aqueous extract of 3R4F smoke were subjected to the analysis.

**Additional file 2: Figure S2.** Comparison of age and pack-years between smokers and COPD subjects. (A) The box plot showing the age and pack-years of non-smokers (NS), smokers (SMK), and COPD subjects (COPD) in publicly available datasets. The box plot presents the median (line) and 25th and 75th percentiles (box); the whiskers are the 5th and 95th percentiles. The dots beyond the whiskers represent outlying data. The histogram shows the number of subjects in each group. Tukey–Kramer multiple comparison analysis revealed differences between NS and SMK (*p < 0.05) and between SMK and COPD (†*p* < 0.05). (2) Correlation between age and pack-years in SMK and COPD.

**Additional file 3: Figure S3.** Correlation analysis of gene expression value of each identified gene with pack-years. Correlation between the pack-years and the normalized intensity value of each gene with all smokers and COPD subjects. The Pearson correlation coefficient (*R*) is shown in the upper right of each image.

**Additional file 4: Figure S4.** Correlation analysis of gene expression value of each identified gene with age. Correlation between the pack-years and the normalized intensity value of each gene with all smokers and COPD subjects. The Pearson correlation coefficient (*R*) is shown in the upper right of each image.

**Additional file 5: Figure S5.** Potential risk factor calculation with in vitro exposure study. The potential risk factor (PRF) index ratios versus control for exposure to the aqueous extract of 3R4F smoke for 4 and 24 h at 0.5, 1.0, and 2.0 cigarettes/L. Each value is presented as the mean and standard deviation of three tissues. Cig: cigarettes.

## Data Availability

Implementations of algorithms and mathematical methods used in the current study are all available as opensource software. Transcriptomics datasets used in current study are available in ArrayExpress at accession number E-MTAB-7992, E-MTAB-1690, E-GEOD-20257, and E-GEOD-8545.

## Abbreviations

3D: Three-dimensional
ALI: Air-liquid interface
AqE: Aqueous extract
COPD: Chronic obstructive pulmonary disease
CS: Cigarette smoke
DEG: Differentially expressed gene
FDR: False discovery rate
MSC: Mesenchymal stem cell
NGP: Next-generation product
PRF: Potential risk factor
RF: Random forest
