# Supplementary figures and images for: Novel biomarker genes which distinguish between smokers and chronic obstructive pulmonary disease patients with machine learning approach
Source: BMC Pulm Med. 2020 Feb 3;20:29. doi: 10.1186/s12890-020-1062-9 (PMC6998147; doi:10.1186/s12890-020-1062-9)

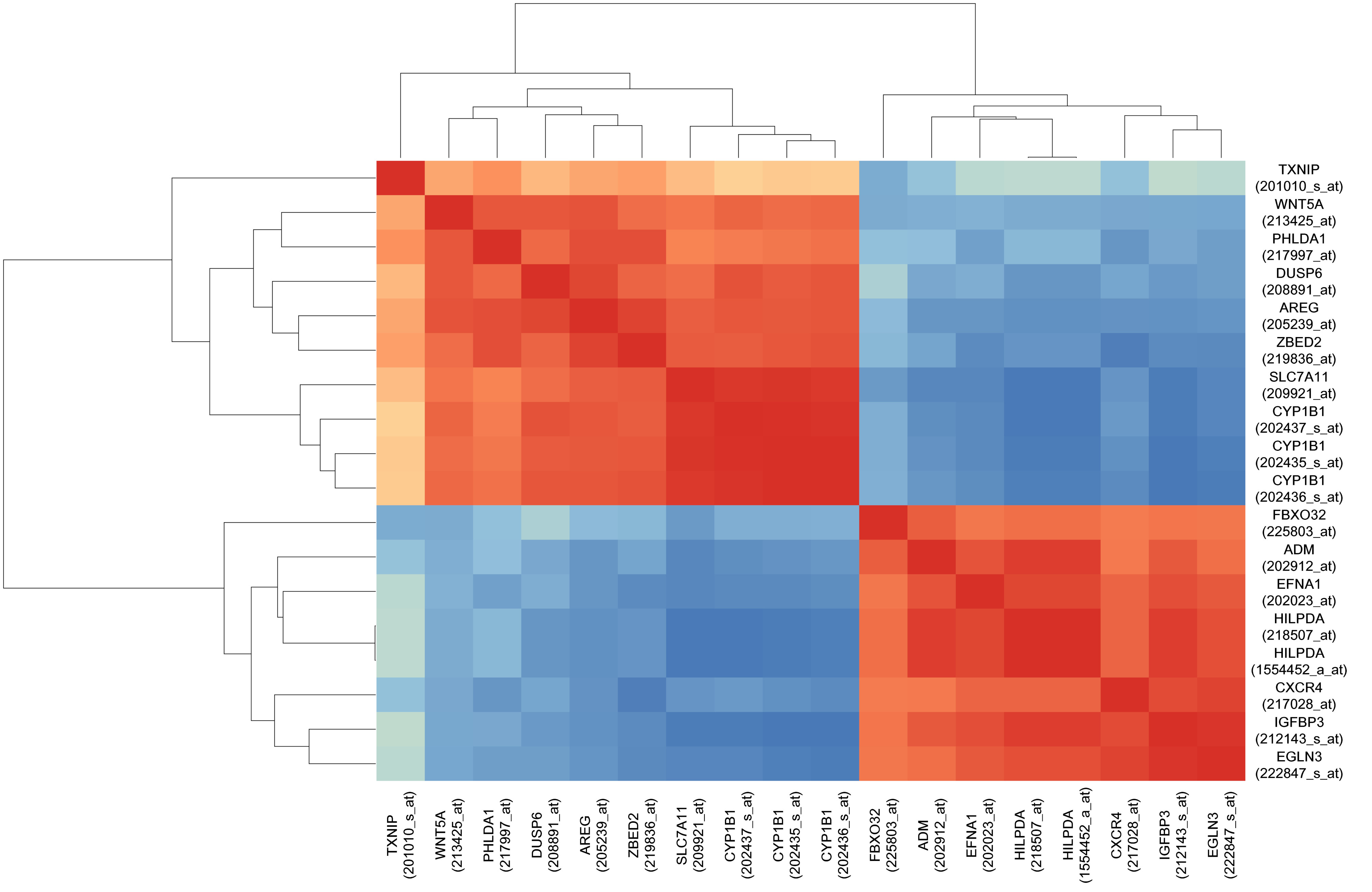

Supplement: Supplementary file 1 — Additional file 1: Figure S1. Spearman’s correlation coefficients analysis. Hierarchical clustering of the Spearman’s correlation coefficients of the 15 identified genes. Normalized intensity values following exposure to the aqueous extract of 3R4F smoke were subjected to the analysis. [file 12890_2020_1062_MOESM1_ESM.png]

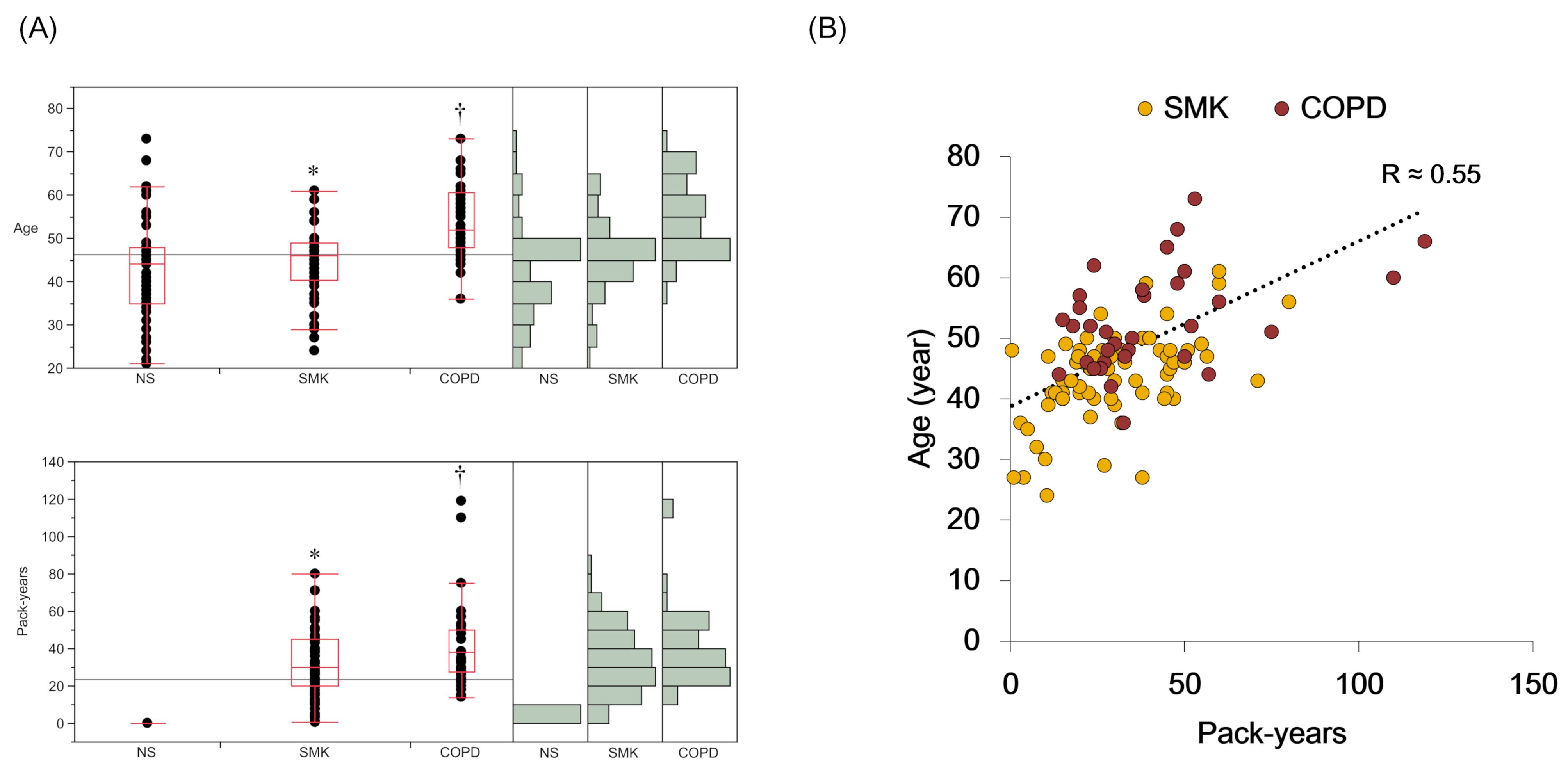

Supplement: Supplementary file 2 — Additional file 2: Figure S2. Comparison of age and pack-years between smokers and COPD subjects. (A) The box plot showing the age and pack-years of non-smokers (NS), smokers (SMK), and COPD subjects (COPD) in publicly available datasets. The box plot presents the median (line) and 25th and 75th percentiles (box); the whiskers are the 5th and 95th percentiles. The dots beyond the whiskers represent outlying data. The histogram shows the number of subjects in each group. Tukey–Kramer multiple comparison analysis revealed differences between NS and SMK (*p < 0.05) and between SMK and COPD (†p < 0.05). (2) Correlation between age and pack-years in SMK and COPD. [file 12890_2020_1062_MOESM2_ESM.png]

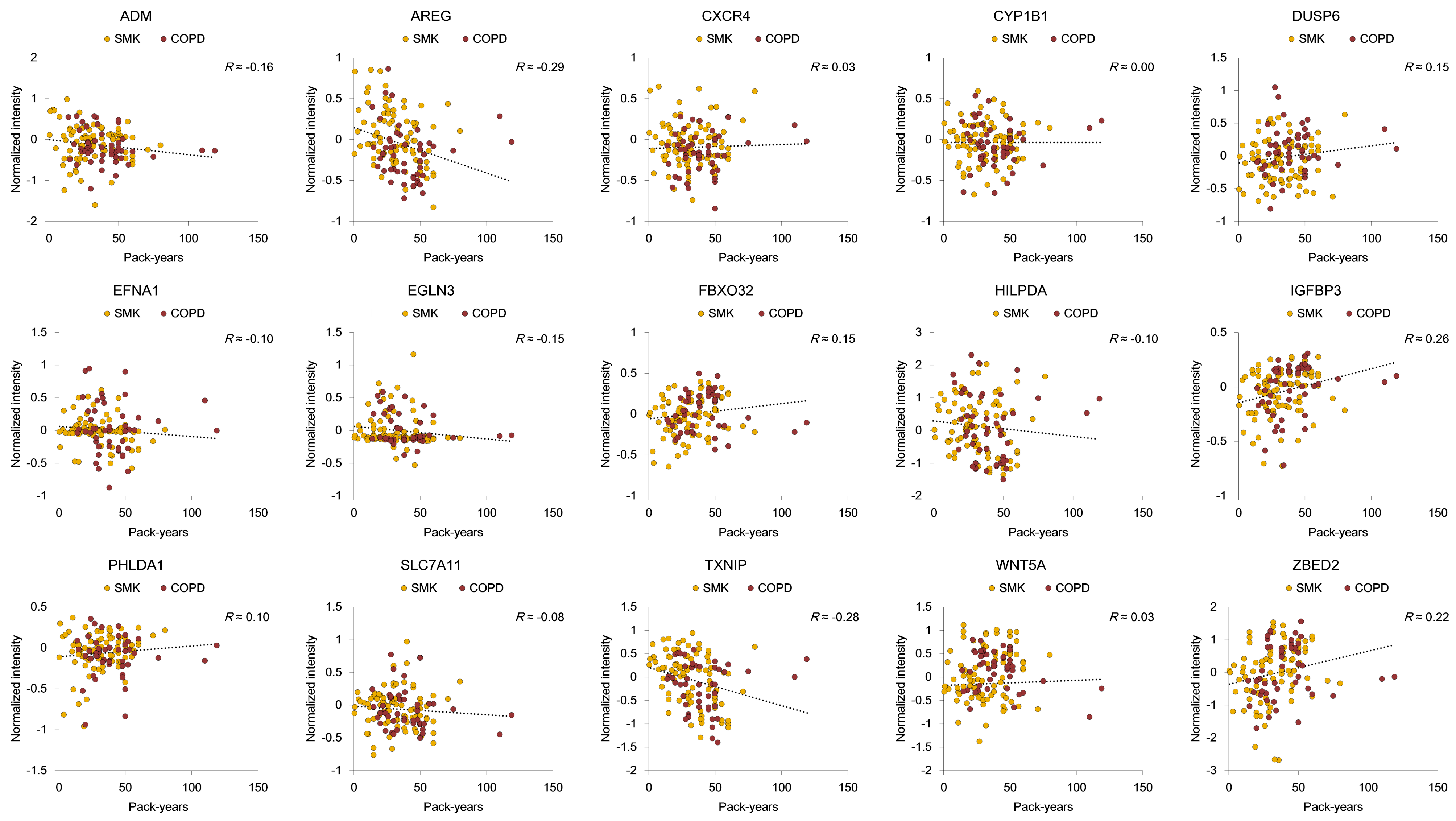

Supplement: Supplementary file 3 — Additional file 3: Figure S3. Correlation analysis of gene expression value of each identified gene with pack-years. Correlation between the pack-years and the normalized intensity value of each gene with all smokers and COPD subjects. The Pearson correlation coefficient (R) is shown in the upper right of each image. [file 12890_2020_1062_MOESM3_ESM.png]

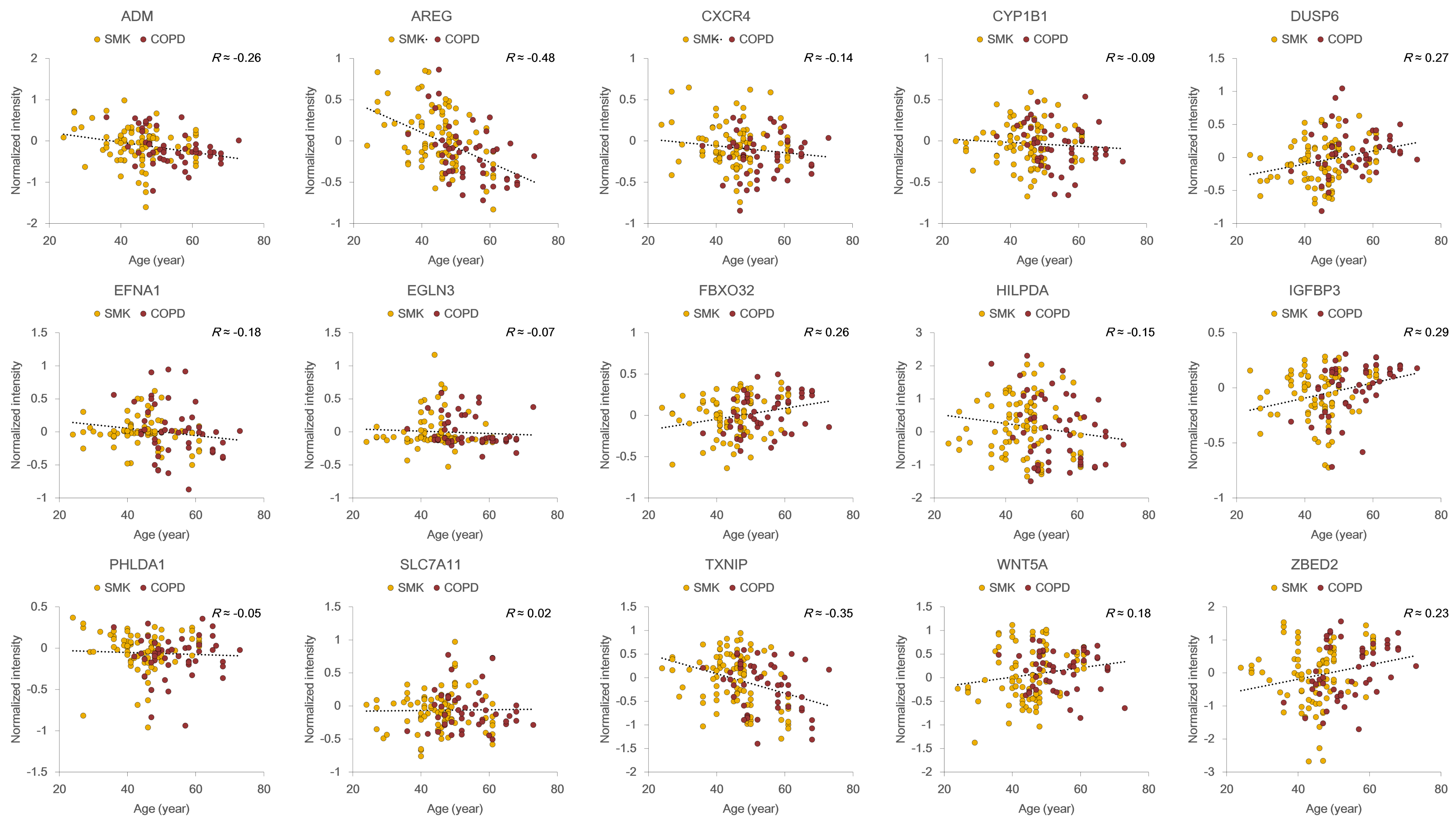

Supplement: Supplementary file 4 — Additional file 4: Figure S4. Correlation analysis of gene expression value of each identified gene with age. Correlation between the pack-years and the normalized intensity value of each gene with all smokers and COPD subjects. The Pearson correlation coefficient (R) is shown in the upper right of each image. [file 12890_2020_1062_MOESM4_ESM.png]

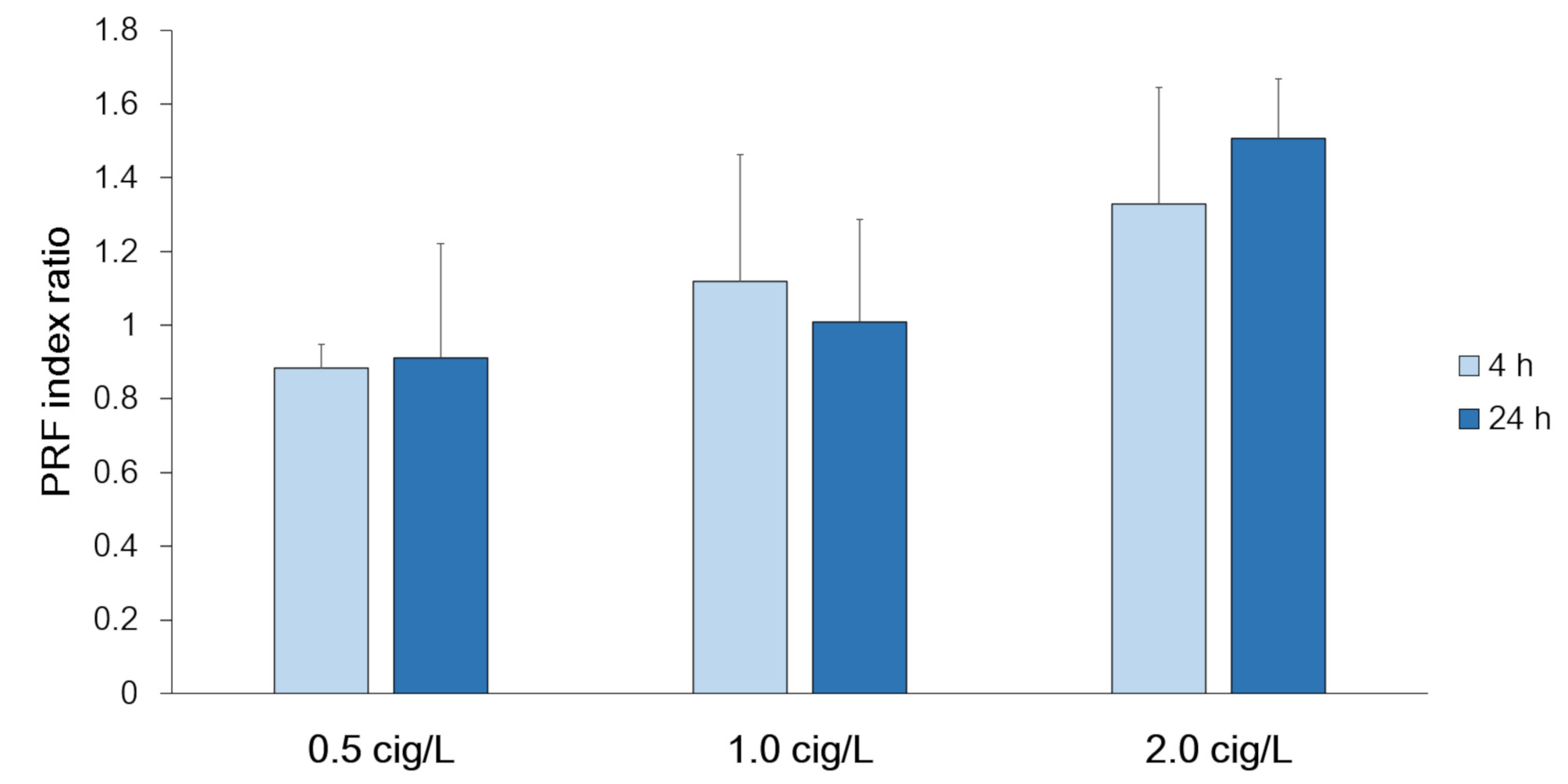

Supplement: Supplementary file 5 — Additional file 5: Figure S5. Potential risk factor calculation with in vitro exposure study. The potential risk factor (PRF) index ratios versus control for exposure to the aqueous extract of 3R4F smoke for 4 and 24 h at 0.5, 1.0, and 2.0 cigarettes/L. Each value is presented as the mean and standard deviation of three tissues. Cig: cigarettes. [file 12890_2020_1062_MOESM5_ESM.png]
